# Establishment of epidemiological cutoff values for clinically relevant *Sporothrix* species using CLSI-broth microdilution

**DOI:** 10.1128/aac.01907-25

**Published:** 2026-04-06

**Authors:** Amanda R. dos Santos, Alexandro Bonifaz, Amanda Bombassaro, Ana Caroline de Sá Machado, Anderson M. Rodrigues, Andrew M. Borman, Arunaloke Chakrabarti, Anuradha Chowdary, Bruno Rediguieri, Elizabeth M. Johnson, Ferry Hagen, Flavio Queiroz-Telles, Gloria M. Gonzalez, Guillermo Garcia-Effron, Isabella Dib Ferreira Gremião, Jacques F. Meis, Juliana Possato Fernandes Takahashi, Luana Pereira Borba dos Santos, Marcia S. C. Melhem, Paola Cappellano, Raimunda Sâmia Nogueira Brilhante, Sandro Antonio Pereira, Sarah Santos Gonçalvez, Sarah Kidd, Sean X. Zhang, Shivaprakash Rudramurthy, Sonia Rozental, Susana Cordoba, Theun De Groot, Wei Liu, Nathan P. Wiederhold, Eelco F. J. Meijer, Shawn R. Lockhart, Philippe J. Dufresne

**Affiliations:** 1 Departamento de Análises Clínicas e Toxicológicas, Faculdade de Ciências Farmacêuticas, Universidade de São Paulo 28133, São Paulo, Brazil; 2 Mycotic Diseases Branch, Centers for Disease Control and Prevention 1242, Atlanta, Georgia, USA; 3 Dermatology Service, Mycology Department, Hospital General de México, "Dr. Eduardo Liceaga" 61575, Mexico City, Mexico; 4 Department of Public Health, Federal University of Paraná 28122, Curitiba, Brazil; 5 Radboudumc-CWZ Center of Expertise for Mycology, Canisius-Wilhelmina Hospital (CWZ) 6034, Nijmegen, the Netherlands,; 6 Laboratório de Pesquisa Clínica em Dermatozoonoses em Animais Domésticos (Lapclin-Dermzoo), Fundação Oswaldo Cruz (Fiocruz), Instituto Nacional de Infectologia Evandro Chagas (INI) 37903 , Rio de Janeiro, Brazil; 7 Department of Microbiology, Immunology, and Parasitology, Discipline of Cellular Biology, Federal University of São Paulo (UNIFESP) 28105, São Paulo, Brazil; 8 National Institute of Science and Technology in Human Pathogenic Fungi 551063 , São Paulo, Brazil; 9 UK Health Security Agency National Mycology Reference Laboratory, Bristol and MRC Centre for Medical Mycology, University of Exeter 3286, Exeter, United Kingdom; 10 Doodhadhari Burfani Hospital and Research Institute, Haridwar, India; 11 Medical Mycology Unit, Department of Microbiology, Vallabhbhai Patel Chest Institute, University of Delhi 72916 , New Delhi, India; 12 Department of Pathology, Universidade Federal do Espírito Santo (UFES) 28126, Vitória-ES, Brazil; 13 Department of Medical Microbiology, University Medical Center Utrecht 647217, Utrecht, the Netherlands; 14 Faculty of Medicine, Autonomous University of Nuevo León 103563 , Monterrey, Mexico; 15 Consejo Nacional de Investigaciones Científicas y Tecnológicas (CONICET), Universidad Nacional del Litoral, Santa Fe de la Vera Cruz 28247, Santa Fe, Argentina; 16 Institute of Translational Research, Cologne, Excellence Center for Medical Mycology (ECMM), University of Cologne 14309, Cologne, Germany; 17 Centro de Patologia, Núcleo de Patologia Quantitativa, Instituto Adolfo Lutz 89119, São Paulo, Brazil; 18 Laboratório de Biologia Celular de Fungos, Centro de Pesquisas em Medicina de Precisão, Universidade Federal do Rio de Janeiro (UFRJ) 28125 , Rio de Janeiro, Brazil; 19 Faculdade de Medicina, Universidade Federal do Mato Grosso do Sul 54534, Campo Grande, Mato Grosso do Sul, Brazil; 20 Laboratório de ainvestigação Médica, Hospital das Clinicas, Faculdade de Medicina, Universidade de São Paulo 28133, São Paulo, Brazil,; 21 Microbiology Section, Grupo Fleury 89577, São Paulo, São Paulo, Brazil; 22 Department of Pathology and Legal Medicine, School of Medicine, One Health Microbiology Laboratory, Postgraduate Program in Medical Microbiology, Postgraduate Program in Medical Sciences, Federal University of Ceará 28121, Fortaleza, Ceará, Brazil; 23 National Mycology Reference Centre, SA Pathology 1066, Adelaide, Australia; 24 Department of Pathology, Johns Hopkins University School of Medicine & Johns Hopkins Hospital 1500 , Baltimore, Maryland, USA; 25 Department of Medical Microbiology, Post Graduate Institute of Medical Education & Research 29751 , Chandigarh, India; 26 Mycology Department of INEIA ANLIS “Dr. C. G. Malbran”, Buenos Aires, Argentina; 27 Peking University First Hospital 26447, Beijing, China; 28 Department of Pathology and Laboratory Medicine, University of Texas Health Science Center at San Antonio 14742, San Antonio, Texas, USA; 29 Laboratoire de Santé Publique du Québec, Institut National de Santé Publique du Québec (INSPQ), Sainte-Anne-de-Bellevue 54470, Québec, Canada; University Children's Hospital Münster, Münster, Germany

**Keywords:** *Sporothrix *spp., antifungal susceptibility testing, zoonotic, epidemiological cutoff values (ECV), sporotrichosis

## Abstract

Sporotrichosis is a globally distributed subcutaneous mycosis caused mainly by *Sporothrix brasiliensis*, *S. schenckii*, and *S. globosa*. Cat-transmitted sporotrichosis, primarily caused by *S. brasiliensis* in South America and to a lesser extent by *S. schenckii* in Southeast Asia, is emerging as a substantial public health concern due to its outbreak potential. Itraconazole is the first-line drug for the treatment of humans and cats, but reduced susceptibility has been reported based on previously proposed epidemiological cut-off values (ECVs). To support resistance surveillance, we aimed to establish the Clinical and Laboratory Standards Institute (CLSI)-endorsed ECVs for these clinically relevant *Sporothrix* species. A total of 3,504 minimum inhibitory concentration (MIC) values for six antifungal agents (amphotericin B, itraconazole, posaconazole, voriconazole, isavuconazole, and terbinafine) were obtained from 19 international laboratories. Four of seven antifungals met the CLSI M57 guidelines criteria to determine the ECV. Established ECVs for amphotericin B were found to be high, with 8 µg/mL for *S. brasiliensis* and *S. globosa*, and 4 µg/mL for *S. schenckii*. Itraconazole ECVs were 4 µg/mL for *S. brasiliensis* and *S. schenckii*. Posaconazole ECVs were 4 µg/mL for all three species (tentative for *S. globosa*), while the terbinafine ECV for *S. brasiliensis* was 0.12 µg/mL. Overall, this study establishes validated ECVs for key antifungals against *Sporothrix* species and identifies a low prevalence of non-wild-type (NWT) isolates (<10% except for *S. schenckii* and posaconazole), supporting ongoing antifungal resistance monitoring.

## INTRODUCTION

Sporotrichosis is a ubiquitous cutaneous/subcutaneous mycosis caused by pathogenic species of the *Sporothrix* genus (1). It is prevalent in tropical and subtropical areas, where infections mostly occur by percutaneous inoculation from plant material containing *Sporothrix* spp., the classical form of the disease (2). Outside South America, this is the common form of the disease and is mainly caused by *S. schenckii* and *S. globosa* (3). In the last three decades, a new species in the *Sporothrix* pathogenic clade*, S. brasiliensis*, has emerged in Brazil and thereafter in other countries in South America (4). *S. brasiliensis* causes large outbreaks in the cat population and is increasingly transmitted to humans by direct and indirect contact with infected cats’ secretions (4). In humans, the usual clinical presentations of sporotrichosis are fixed cutaneous or lymphocutaneous and multifocal disseminated infections in HIV patients. Other clinical presentations, including osteoarticular, pulmonary, and multifocal infections in non-immunocompromised patients, are relatively rare (5, 6). In cats, the most prevalent clinical presentation is the disseminated cutaneous form, typically associated with extracutaneous manifestations, including respiratory involvement and lymphadenomegaly (7, 8). Remarkably, in the last decade, there have also been several reports of cat-transmitted sporotrichosis in Thailand and Malaysia, which were caused by *S. schenckii* (9–11).

In South America, and to a lesser extent in Southeast Asia, zoonotic sporotrichosis has become a major public health problem because of its capacity to rapidly spread and its epidemic potential (12). Currently, there is no vaccine against sporotrichosis, and the treatment of infected cats is the main prevention strategy to prevent outbreaks and further spread of the disease (13). There are limited antifungal treatment options in cats and humans. Itraconazole is the first-choice antifungal drug for human disease, while terbinafine and potassium iodide are used as alternatives (14). Amphotericin B is used for severe human infections (15, 16). In feline sporotrichosis, itraconazole, administered either as monotherapy or in combination with potassium iodide, remains the first-line drug. Deoxycholate amphotericin B can be administered by local injections in the lesion in cases refractory to itraconazole (17). Terbinafine demonstrates limited antifungal effectiveness (18). Additionally, there are a few documented cases reporting successful use of posaconazole and isavuconazole in treatment-refractory infections (19, 20). In Brazil, effective treatment of feline sporotrichosis is hindered by multiple factors, including owner treatment non-adherence, socio-economic constraints, the long duration of therapy, challenges with the antifungal administration to cats, and the cost of itraconazole, which is not provided free of charge in many public health services (21). Treatment abandonment has been reported in 34%–39% of infected cats (22, 23). Poor treatment compliance and treatment failure in cats may have an impact on human health, as it can lead to increased fungal burden, antifungal exposure, and could favor the development of antimicrobial resistance. Reduced susceptibility of *S. brasiliensis* to itraconazole was suggested in a human case non-responsive to itraconazole (24). As cats are treated with the same antifungal agents used in humans, the potential emergence of antimicrobial resistance may represent an additional challenge for the management of human sporotrichosis.

The detection of isolates with non-wild-type (NWT) minimal inhibitory concentrations (MICs) enables surveillance of the emergence and spread of antifungal resistance (25). Establishing a baseline of MICs and associated clinical presentations is the first step for monitoring the emergence of resistance. For this purpose, antifungal breakpoints for medically relevant *Sporothrix* species are necessary. Owing to a lack of studies reporting MICs along with clinical data and treatment outcomes, breakpoints have not been established, nor are official epidemiological cut-off values (ECVs) available, which enable the identification of isolates with reduced susceptibility and potential antifungal resistance mechanisms.

Several studies have reported azole MIC values for *S. brasiliensis* (26–31), whereas a multicenter international study proposed ECVs for *S. brasiliensis* and *S. schenckii* to azoles and amphotericin B, and to terbinafine for *S. brasiliensis* only (27). Although this study included a high number of MIC values, results were not submitted for approval and validation to the Clinical and Laboratory Standards Institute (CLSI). Here, using an expanded contemporary data set from a total of 19 participating laboratories, including some of the MIC values presented in the previous ECV study (27), we established MIC distributions, ECVs, percent NWT, modal MICs, MIC_90_s, and geometric mean MICs for six antifungals, including amphotericin B, terbinafine, and triazoles (itraconazole, posaconazole, voriconazole, and isavuconazole), for *S. schenckii, S. globosa,* and/or *S. brasiliensis*.

## RESULTS

A total of 3,504 MIC values for *S. brasiliensis* (*n* = 1,742), *S. globosa* (*n* = 548), and *S. schenckii* (*n* = 1,214) isolates were obtained from 19 laboratories that performed antifungal susceptibility testing (AFST) using the broth microdilution method as outlined in the CLSI reference standard M38 Ed3 for filamentous fungi (32).

**Fig 1:**
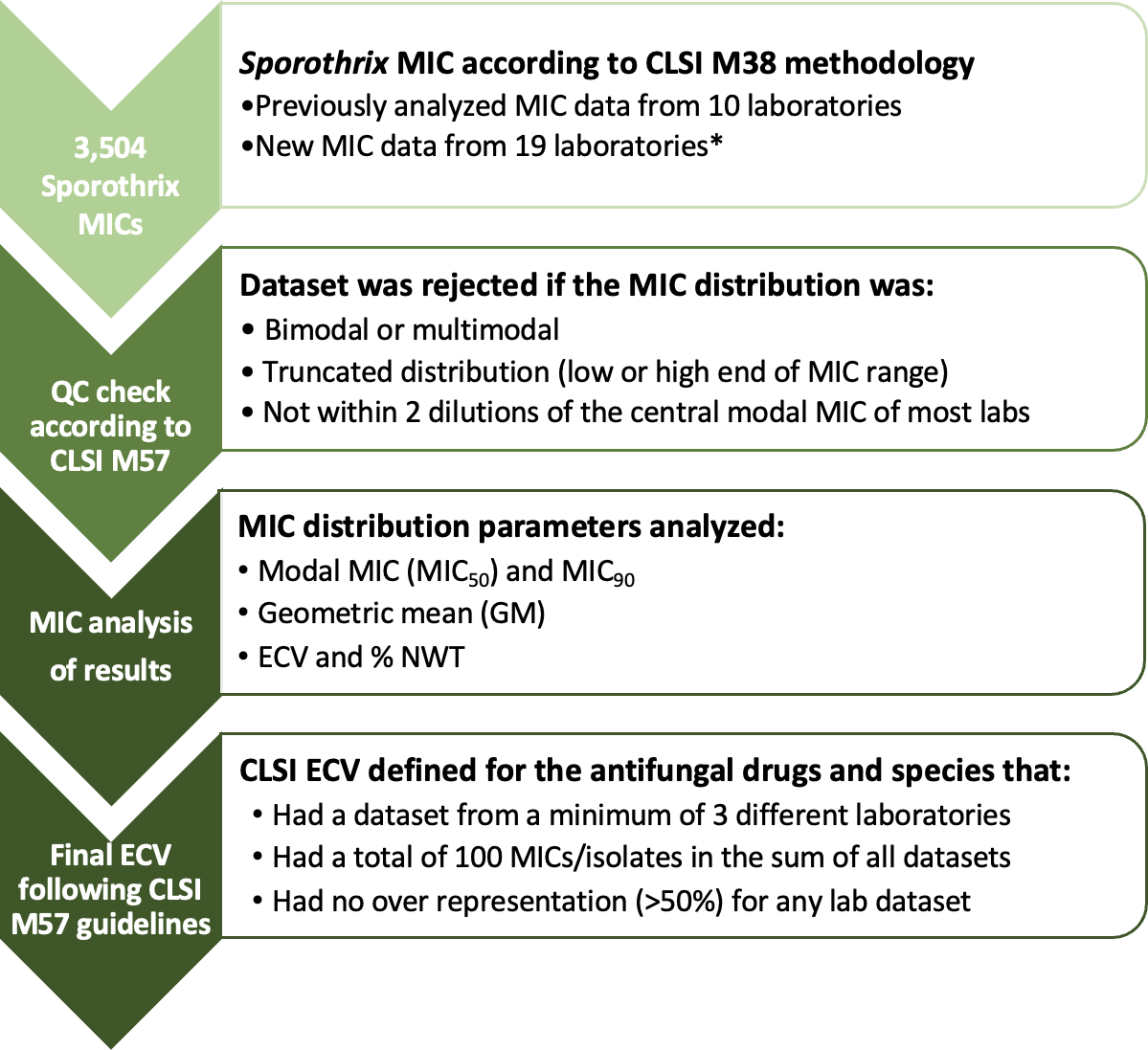
Flow chart showing the analysis steps and the inclusion and exclusion criteria for received MIC data sets. A total of 3,504 MIC data for *S. brasiliensis*, *S. globosa*, and *S. schenckii* isolates were obtained from 19 laboratories that performed AFST using the broth M38 microdilution method. MIC data sets were rejected if they did not conform to CLSI M57 guidelines (such as being multimodal, truncated at the low or high end of the tested range, or not within two dilutions of the central modal MIC of most laboratories). After rejection of the MIC data sets, MIC distributions were determined for amphotericin B, terbinafine, and triazoles, together with the geometric mean MIC, MIC_50_, and MIC_90_ data. *A subset of the MICs (up to 2017) came from nine laboratories involved in a previous study (27). MIC, minimum inhibitory concentration; CLSI, Clinical and Laboratory Standards Institute; ECV, epidemiological cut-off value; NWT, non-wild type.

The analysis steps and the exclusion criteria used for received MIC data sets are summarized in Fig. 1. A subset of the MICs (up to 2017) came from nine laboratories involved in a previous study (27). Additional contemporary MIC data sets were also received from some of those laboratories, as well as from 10 new sites. MIC distributions were determined for amphotericin B (Table 1), terbinafine (Table 2), and triazoles (Table 3), together with the geometric mean MIC, MIC_50_, and MIC_90_ data. The number of isolates and participating laboratories varied across antifungals, as did the number of isolates among the three species (Tables 1 to 4).

**TABLE 1:** Amphotericin B MIC distribution for *Sporothrix* species^*a*^

Antifungal	Labs	Isolates	MIC values (µg/mL)											GM	MIC_50_	MIC_90_
			≤0.03	0.06	0.12	0.25	0.5	1	2	4	8	16	>16			
*S. brasiliensis*	7	212	6	0	7	18	23	**70**	68	19	1	0	0	0.99	1	4
*S. globosa*	10	137	0	0	13	4	9	19	**49**	33	6	1	3	2.03	2	4
*S. schenckii*	9	176	2	0	5	11	18	**75**	43	18	1	1	2	1.13	1	4

^*a*^ Modes in bold.

**TABLE 2:** Terbinafine MIC distributions for *Sporothrix* species^*a*^^,^^*b*^

Antifungal	Labs	Isolates	MIC values (µg/mL)												GM	MIC_50_	MIC_90_
			0.004	0.008	0.016	0.03	0.06	0.12	0.25	0.5	1	2	4	8			
																	
Terbinafine																	
*S. brasiliensis*	4	270	1	5	9	87	**129**	27	6	6	0	0	0	0	0.05	0.06	0.12
*S. globosa*	3	38	0	0	0	0	4	0	2	15	**17**	0	0	0	0.53	0.5	1
*S. schenckii*	3	42	0	0	0	1	11	**15**	8	7	0	0	0	0	0.14	0.12	0.5

^*a*^ Modes in bold.

^*b*^ GM, geometric mean; MIC, minimum inhibitory concentration; MIC_50_ or MIC_90_, MIC that inhibits 50% or 90% of the isolates tested.

**TABLE 3:** Triazole MIC distributions for *Sporothrix* species^*a*^^,^^*b*^

Antifungal	Labs	Isolates	MIC values (µg/mL)												GM	MIC_50_	MIC_90_
			≤0.016	0.03	0.06	0.12	0.25	0.5	1	2	4	8	16	>16			
Isavuconazole																	
*S. brasiliensis*	3	93	0	0	1	1	2	1	9	14	20	**35**	9	1	4.06	4	>16
*S. globosa*	3	20	0	0	0	0	0	0	0	1	2	4	**11**	2	11.71	16	>16
*S. schenckii*	5	74	0	0	0	0	0	0	10	5	18	**26**	8	7	5.71	8	>16
Itraconazole																	
*S. brasiliensis*	7	216	5	1	3	14	35	50	**69**	29	2	0	0	8	0.65	1	2
*S. globosa*	10	96	1	13	4	9	15	**16**	9	12	3	4	1	9	0.55	0.5	>16
*S. schenckii*	11	242	0	2	6	10	31	**67**	63	38	5	2	1	17	0.86	1	4
Posaconazole																	
*S. brasiliensis*	6	291	0	0	2	4	13	58	**100**	**100**	11	0	0	3	1.08	1	2
*S. globosa*	8	80	0	1	0	3	14	19	**27**	8	2	0	1	5	0.69	1	4
*S. schenckii*	10	266	0	0	1	10	14	54	**83**	41	11	9	8	35	1.57	1	>16
Voriconazole																	
*S. brasiliensis*	5	137	0	0	0	0	0	2	6	6	16	41	**54**	12	9.03	8	16
*S. globosa*	9	111	0	11	0	6	2	3	3	4	5	15	22	**40**	9.29	16	>16
*S. schenckii*	10	266	0	4	0	0	1	4	13	27	43	65	**86**	23	6.83	8	16

^*a*^ Modes in bold.

^*b*^ GM, geometric mean; MIC, minimum inhibitory concentration; MIC_50_ or MIC_90_, MIC that inhibits 50% or 90% of the isolates tested.

**TABLE 4:** Epidemiological cutoff values (ECV) for *Sporothrix* species and % non-wild type (NWT)^*a*^

Species	Labs included out of total	Isolates included out of total	ECV	% NWT
Amphotericin B				
*S. brasiliensis*	7/9 (78%)	212/370 (57%)	8^*c*^	0%
*S. globosa*	10/10 (100%)	137 (100%)	8^*c*^	3.1%
*S. schenckii*	9/13 (69%)	176/280 (63%)	4^*c*^	2.3%
Itraconazole				
*S. brasiliensis*	7/10 (70%)	216/428 (51%)	4^*c*^	3.7%
*S. globosa*	10/11 (91%)	96/129 (74%)	ILV	–
*S. schenckii*	11/13 (85%)	242/286 (85%)	4^*c*^	8.3%
Isavuconazole				
*S. brasiliensis*	3/4 (75%)	93/154 (60%)	TR-H	–
*S. globosa*	3/3 (100%)	20 (100%)	TR-H	–
*S. schenckii*	5/5 (100%)	74 (100%)	TR-H	–
Posaconazole				
*S. brasiliensis*	7/9 (78%)	291/331 (88%)	4^*c*^	1.0%
*S. globosa*	8/9 (89%)	80/113 (71%)	(4)^*c*^	7.5%
*S. schenckii*	10/10 (100%)	266 (100%)	4^*c*^	19.5%
Voriconazole				
*S. brasiliensis*	5/6 (83%)	137/189 (100%)	TR-H	–
*S. globosa*	9/9 (100%)	111 (100%)	TR-H	–
*S. schenckii*	10/10 (100%)	266 (100%)	TR-H	–
Terbinafine				
*S. brasiliensis*	4/4 (100%)	270 (100%)	0.12	4.4%
*S. globosa*	3/3 (100%)	38 (100%)	(2^*b*^)	0%
*S. schenckii*	3/3 (100%)	42 (100%)	(0.5^*b*^)	0%

^*a*^ –, ECV not defined; TR-H, ECV cannot be defined for this species-antifungal combination because the MIC distribution is truncated at the high end of the recommended testing range. ILV, ECV cannot be defined for this species-antifungal combination because of interlab variation.

^*b*^ Tentative ECVs are presented in parentheses (the number of isolates is <100 and >35).

^*c*^ The ECV is high which indicates limited *in vitro* susceptibility to this agent. An MIC lower than the ECV does not imply that the isolate is susceptible to that antifungal agent.

MIC data sets were rejected if they did not conform to CLSI M57 guidelines (such as being multimodal, truncated at the highest or lowest value, or not within two dilutions of the central modal MIC of most laboratories). Greater interlaboratory variation and rejected laboratory MIC data sets were seen with *S. brasiliensis* for amphotericin B (22%) and azoles (17%–30%), and *S. schenckii* with amphotericin B (31%) (Table 4). For all other species/antifungal combinations, the percentage of MIC data sets from laboratories included was ≥85%.

Data for four of six antifungals met the CLSI M57 guidelines (minimum of 3 different laboratories and 100 isolates) for determination of ECVs via the iterative statistical method with ECOFFinder (V2.1). Tentative ECVs were also calculated if the number of isolates was insufficient (<100 and >35 isolates). For amphotericin B, ECVs were 8 µg/mL for *S. brasiliensis* and *S. globosa*, and 4 µg/mL for *S. schenckii*. ECVs for itraconazole and posaconazole were 4 µg/mL for *S. brasiliensis* and *S. schenckii*, while a tentative ECV of 4 µg/mL was calculated for *S. globosa* and posaconazole. Finally, the ECVs for terbinafine were 0.12 µg/mL for *S. brasiliensis*, and only tentative ECVs for *S. globosa* (2 µg/mL) and *S. schenckii* (0.5 µg/mL) could be defined due to a lack of isolates (Table 4). The itraconazole ECV for *S. globosa* could not be determined due to a high variability in MICs among different laboratories. Additionally, the ECVs for isavuconazole and voriconazole could not be established, as MICs were truncated on the high end of the recommended testing range.

The percentage of NWT isolates with MICs above the established ECV was determined for each drug and species. For amphotericin B and terbinafine, the percentage of NWT strains was <5% for all species. The percentage of NWT strains was <5% for itraconazole and posaconazole against *S. brasiliensis* isolates. In contrast, 8.3% and 19.5% of *S. schenckii* isolates were NWT for itraconazole and posaconazole, respectively, whereas 7.5% of *S. globosa* isolates were NWT for posaconazole (Table 4).

## DISCUSSION

In this study, we established ECVs for the filamentous phase of *S. brasiliensis*, *S. schenckii,* and *S. globosa* for the antifungal drugs amphotericin B, itraconazole, terbinafine, and posaconazole following CLSI M57 guidelines. These ECVs were generated with a contemporary MIC data set and accepted by the CLSI antifungal subcommittee. For terbinafine and posaconazole, tentative ECVs were proposed for *S. globosa* and/or *S. schenckii*.

A previous multicenter study (27) proposed tentative *S. brasiliensis* ECVs for itraconazole, terbinafine, and amphotericin B of 2, 0.12, and 4 µg/mL, respectively. The established ECVs defined here were one dilution higher for itraconazole (4 µg/mL) and amphotericin B (8 µg/mL), whereas for terbinafine the ECV remained unchanged. Additionally, the tentative *S. schenckii* ECVs of itraconazole (4 µg/mL) and amphotericin B (4 µg/mL) were identical to the ECVs in the present evaluation. For *S. globosa,* no tentative ECVs have previously been proposed. The established ECV values for itraconazole (4 µg/mL) and amphotericin B (4 to 8 µg/mL) for the three pathogenic *Sporothrix* species are relatively high compared to most clinically significant molds, such as *A. fumigatus* (32). Some, however, including *Fusarium* and *Scedosporium* spp. and the Mucorales, are known to have relatively high MICs for these agents.

Importantly, ECVs are intended to distinguish NWT isolates that may harbor resistance mechanisms from wild-type populations of a species. They are solely derived from the natural distribution of MICs and do not incorporate pharmacokinetic/pharmacodynamic parameters or clinical outcome data. As such, they do not predict the therapeutic response; an MIC below the ECV does not necessarily indicate susceptibility to that antifungal agent and clinical success, while an MIC above the ECV does not automatically imply resistance and expected treatment failure. With regard to sporotrichosis, high MICs against itraconazole, based on the previously suggested tentative ECV (2 µg/mL), were not found to correlate with antifungal treatment failure or clinical outcome in humans and cats (23, 26, 31). In cats, none of the 47 feline isolates from Rio de Janeiro exhibited high MICs, and no association with clinical outcome was observed (23), whereas at the Brazil–Argentina border, therapeutic failure occurred despite low initial MICs (31). This could be a reflection of the pharmacokinetics of itraconazole, which achieves high concentrations in the skin, the usual site of infection. Nonetheless, in other reports on *S. brasiliensis* isolates from cats and humans, high MICs (4 to >16 µg/mL) were reported in refractory or more severe cases (24, 28, 33–35).

Using these newly defined ECVs, we found that the percentage of NWT isolates varied by fungal species and antifungal drug. The percentage of NWT isolates was low (<5%) for amphotericin B and terbinafine for all three pathogenic *Sporothrix* species and was also low for itraconazole and posaconazole for *S. brasiliensis*. Higher percentages of NWT isolates (>5%) were found for *S. schenckii* with 8.3% and 19.5% for itraconazole and posaconazole, respectively, and for *S. globosa* with 7.5% NWT isolates according to tentative ECV for posaconazole. Another study also reported higher itraconazole and posaconazole MICs for *S. schenckii* than for *S. brasiliensis* (36). The NWT isolates, especially those found among *S. schenckii* and *S. globosa,* may harbor resistance mechanisms as observed for *A. fumigatus* (37) and *Trichophyton indotineae* (38). However, genes linked to resistance mechanisms for *Sporothrix* species are poorly investigated. Resistance has been correlated with substitutions of the *CYP51* and/or *TAC1* genes in a few *S. brasiliensis* isolates (29, 39), but there are no resistance mechanisms reported that are correlated with the high MIC values of *S. schenckii* or *S. globosa*.

No ECV could be established for isavuconazole or voriconazole, drugs that share a similar structure and activity profile, as MICs were truncated at the high end of the recommended testing range, which suggests potential intrinsic resistance or reduced susceptibility. These results are consistent with the guideline recommendations against the use of isavuconazole and voriconazole for sporotrichosis treatment (14, 15), which are based on the lack of efficacy of these drugs *in vitro* (34, 40) and in mice (41).

*Sporothrix* spp. are thermally dimorphic fungi and, depending on incubation temperatures of 35°C or 30°C, grow in either the yeast or the mycelial phase, respectively. This is important in the context of AFST protocols because conidia are used for inoculation, but the temperature of incubation is conductive to yeast growth. In this study, the CLSI M38 methodology for the mycelial phase was used. Some studies have demonstrated that MIC values of the same isolate were ≥8-fold lower in the yeast phase when compared to the mold phase (24, 27, 30, 31, 34). To obtain a *Sporothrix* culture with a pure yeast phase, a long incubation time (>2 weeks) at 35°C is needed and confirmation by microscopy is essential (31). A pure mycelial phase is easier to obtain by culturing at 30°C for 1 week. There is no consensus among experts on whether the establishment of ECVs should be based on the mold or yeast phase. While some researchers suggest that the ECVs should be based on the yeast form, as this phase is the pathogenic infecting form in the host and might therefore better reflect the clinical outcome (34), others highlight the impractical time to results needed for conversion to the yeast form, which can take up to 2 weeks. Mold phase testing is easier to standardize among different laboratories, which is fundamental for establishing ECV. We also found that the MIC data were more similar among the different participant laboratories with the mycelial phase as compared to the yeast phase (data not shown).

There is overall agreement that the current *Sporothrix* CLSI mold phase protocol CLSI M38 (32) needs some modifications, since testing at 35°C may lead to a mix of yeast and mold phases. In this study, we found a high level of interlaboratory variation for some antifungals, with a modal MIC not within one or two dilutions to the central modal MIC, leading to rejection of MIC data from different laboratories, especially for *S. brasiliensis* for which 17% to 30% of laboratories excluded amphotericin B and azoles data and for *S. schenckii* for which 31% of labs excluded amphotericin B data. This inter-laboratory variation could be partially explained by the mix of mold and yeast phases, with the yeast phase decreasing the MIC for many antifungals, including itraconazole (34). A solution for this issue would be to revise the CLSI protocol to adjust the incubation temperature to 30°C to ensure that there are no mixed forms for a potentially more standardized, less variable MIC assay. In that case, the ECVs presented here would need to be reevaluated. In addition, it would be imperative to distribute *Sporothrix* spp. reference quality control (QC) isolates with defined MICs to allow laboratories comparative assessment of the standardized method. Unfortunately, there are currently no *Sporothrix* QC strains listed in M38M51S. Further efforts should be made to establish *Sporothrix* QC strains with defined MIC ranges for the three *Sporothrix* species, which should be made available to allow thorough verification and validation of the CLSI broth microdilution methodology for *Sporothrix* species.

A limitation of this study is the inability to establish ECVs for all antifungal drugs evaluated here across the three main pathogenic species of *Sporothrix*. For *S. globosa,* ECVs could not be established for all drugs, due to high interlaboratory variation of MICs and many high MIC values. Another limitation is the lack of knowledge regarding the potential presence of both yeast and mold phases, following the CLSI M38 protocol, and whether that caused the high interlaboratory variations. Future studies at different mycology laboratories should test and validate changes in the CLSI M38 protocol to test *Sporothrix* in the pure mycelial phase and establish well-characterized *Sporothrix* QC strains to overcome current challenges. MICs of more isolates are needed to confirm the tentative ECVs found here for terbinafine against *S. schenckii* and *S. globosa*.

In summary, following the CLSI M57 methodology for the mycelial phase, we have established official ECVs for *S. brasiliensis* to itraconazole, posaconazole, terbinafine, and amphotericin B; for *S. schenckii* to itraconazole, posaconazole, and amphotericin B; and a single ECV for *S. globosa* against amphotericin B. These ECVs will facilitate the tracking of NWT strains in the current epidemic in Brazil, and emerging clusters in Southeast Asia. The establishment of an ECV is also the first step toward the systematic collection of laboratory data and possible correlations with clinical data to set antifungal breakpoints.

## MATERIALS AND METHODS

### Experimental design

Laboratories known to test antifungal susceptibility using the CLSI methodologies were contacted to request MIC data for clinically relevant *Sporothrix* species (*S. brasiliensis, S. globosa,* and *S. schenckii*). Inclusion criteria were that species identification was performed using molecular techniques and AFST using the broth microdilution method as outlined in the CLSI reference standard M38 Ed3 for filamentous fungi. MIC data for *S. brasiliensis*, *S. globosa*, and *S. schenckii* isolates were obtained from 19 laboratories from 10 countries on five continents and included in the analysis (Asia: China and India; Europe: Netherlands and United Kingdom; North America: Canada, Mexico, and USA; South America: Argentina and Brazil; Oceania: Australia). The detailed listing of the 19 laboratories is provided as supplementary material (Table S1).

### Antifungal susceptibility testing

AFST was performed by broth microdilution as outlined in the CLSI reference standard M38 Ed3 for filamentous fungi (32). The isolates used as reference or quality control strains were *Aspergillus flavus* ATCC 204304, *Aspergillus fumigatus* ATCC 204305, *Aspergillus fumigatus* ATCC MYA 3626, *Aspergillus fumigatus* ATCC MYA 3627, *Aspergillus fumigatus* NCPF 7097, *Aspergillus fumigatus* NCPF 7100, *Candida parapsilosis* ATCC 22019 (CBS 604), *Candida krusei (Pichia kudriavzevii*) ATCC 6258 (CBS 573), *Hamigera insecticola* ATCC MYA-3630, the quality controls were within M38M51S CLSI ranges. Amphotericin B, isavuconazole, itraconazole, posaconazole, terbinafine, and voriconazole were tested. The MICs were determined visually after 48–72 h (2–3 days, depending on the growth rate of the individual isolate) of incubation at 35°C. The inoculum was adjusted to an absorbance of 530 nm at 0.09–0.13 and verified by hematocytometer counting to 0.2–2.5 × 10^6^ conidia/mL or with a Cellometer X2 (Nexcelom, Manchester, United Kingdom) to perform cell counts and prepare the inoculum. The antifungal susceptibility endpoints for itraconazole, voriconazole, posaconazole, isavuconazole, terbinafine, and amphotericin B were 100% inhibition of growth compared to the drug-free control.

### Epidemiological cutoff values

The ECV was established using the iterative statistical method with ECOFFinder (V2.1) with a 97.5% threshold following CLSI M57 ECV guidelines (32, 42). The percent NWT, modal MIC, MIC_90_, and geometric mean were calculated for each antifungal. Tentative ECVs were calculated when the number of isolates was less than 100 and greater than 35. MIC data sets were rejected if they did not conform to the CLSI M57 guideline (multimodal, truncated, or not within two dilutions of the central modal MIC of most laboratories).

## SUPPLEMENTAL MATERIAL

Table S1 aac.01907-25-s0001.docx List of the 19 laboratories sending MIC data in the five continents.

ASM does not own the copyrights to Supplemental Material that may be linked to, or accessed through, an article. The authors have granted ASM a non-exclusive, world-wide license to publish the Supplemental Material files. Please contact the corresponding author directly for reuse.

## References

[1] Chakrabarti A, Bonifaz A, Gutierrez-Galhardo MC, Mochizuki T, Li S. 2015. Global epidemiology of sporotrichosis. Med Mycol 53:3–14. doi:10.1093/mmy/myu062 25526781

[2] Rodrigues AM, de Hoog GS, de Camargo ZP. 2016. Sporothrix species causing outbreaks in animals and humans driven by animal–animal transmission. PLoS Pathog 12:e1005638. doi:10.1371/journal.ppat.1005638 27415796 PMC4945023

[3] Zhang Y, Hagen F, Stielow B, Rodrigues AM, Samerpitak K, Zhou X, Feng P, Yang L, Chen M, Deng S, Li S, Liao W, Li R, Li F, Meis JF, Guarro J, Teixeira M, Al-Zahrani HS, Pires de Camargo Z, Zhang L, de Hoog GS. 2015. Phylogeography and evolutionary patterns in Sporothrix spanning more than 14 000 human and animal case reports. Persoonia 35:1–20. doi:10.3767/003158515X687416 26823625 PMC4713101

[4] de Carvalho JA, Beale MA, Hagen F, Fisher MC, Kano R, Bonifaz A, Toriello C, Negroni R, Rego RS de M, Gremião IDF, Pereira SA, de Camargo ZP, Rodrigues AM. 2021. Trends in the molecular epidemiology and population genetics of emerging Sporothrix species. Stud Mycol 100:100129. doi:10.1016/j.simyco.2021.100129 35027980 PMC8693333

[5] de Oliveira VF, Petrucci JF, Taborda M, Brener PZ, Kremer PGDBB, Randi BA, Magri ASGK, Magri MMC, Levin AS, Silva GD. 2024. Clinical characteristics, diagnosis, and treatment of central nervous system sporotrichosis: systematic review and meta-analysis. Mycoses 67:e13697. doi:10.1111/myc.13697 38374494

[6] Xavier MO, Poester VR, Trápaga MR, Stevens DA. 2023. Sporothrix brasiliensis: epidemiology, therapy, and recent developments. JoF 9:921. doi:10.3390/jof9090921 37755029 PMC10532502

[7] Pereira SA, Gremião IDF, Kitada AAB, Boechat JS, Viana PG, Schubach TMP. 2014. The epidemiological scenario of feline sporotrichosis in Rio de Janeiro, State of Rio de Janeiro, Brazil. Rev Soc Bras Med Trop 47:392–393. doi:10.1590/0037-8682-0092-2013 25075494

[8] Chacon AFP, Figueiredo ABF, Boechat JS, Reis EG, Honorato CCDS, Corrêa ML, Pereira SA, Gremião IDF. 2025. Prospective uncontrolled interventional study of itraconazole and β-glucans (Euglena gracilis) to assess safeness and clinical effectiveness in cats with cutaneous and mucosal sporotrichosis. Vet Sci 12:830. doi:10.3390/vetsci12090830 41012754 PMC12474118

[9] Jirawattanadon P, Bunyaratavej S, Leeyaphan C, Chongtrakool P, Sitthinamsuwan P, Panjapakkul W, Prasertsook S, Saengthong-Aram P, Wareesawetsuwan N, Posri J, Pattanaprichakul P. 2024. Clinical manifestations, antifungal drug susceptibility, and treatment outcomes for emerging zoonotic cutaneous sporotrichosis, Thailand. Emerg Infect Dis 30:2583–2592. doi:10.3201/eid3012.240467 39592393 PMC11616660

[10] Han HS, Kano R. 2021. Feline sporotrichosis in Asia. Braz J Microbiol 52:125–134. doi:10.1007/s42770-020-00274-5 32363567 PMC7966660

[11] Siew HH. 2017. The current status of feline sporotrichosis in Malaysia. Med Mycol J 58:E107–E113. doi:10.3314/mmj.17.014 28855477

[12] Ribeiro dos Santos A, Misas E, Min B, Le N, Bagal UR, Parnell LA, Sexton DJ, Lockhart SR, de Souza Carvalho Melhem M, Takahashi JPF, Oliboni GM, Bonfieti LX, Cappellano P, Sampaio JLM, Araujo LS, Alves Filho HL, Venturini J, Chiller TM, Litvintseva AP, Chow NA. 2024. Emergence of zoonotic sporotrichosis in Brazil: a genomic epidemiology study. The Lancet Microbe 5:e282–e290. doi:10.1016/S2666-5247(23)00364-6 38432234 PMC11487493

[13] Rossow JA, Queiroz-Telles F, Caceres DH, Beer KD, Jackson BR, Pereira JG, Ferreira Gremião ID, Pereira SA. 2020. A one health approach to combatting Sporothrix brasiliensis: narrative review of an emerging zoonotic fungal pathogen in south America. J Fungi (Basel) 6:247. doi:10.3390/jof6040247 33114609 PMC7712324

[14] Thompson GR III, Le T, Chindamporn A, Kauffman CA, Alastruey-Izquierdo A, Ampel NM, Andes DR, Armstrong-James D, Ayanlowo O, Baddley JW, et al.. 2021. Global guideline for the diagnosis and management of the endemic mycoses: an initiative of the European confederation of medical mycology in cooperation with the international society for human and animal mycology. Lancet Infect Dis 21:e364–e374. doi:10.1016/S1473-3099(21)00191-2 34364529 PMC9450022

[15] Kauffman CA, Bustamante B, Chapman SW, Pappas PG, Infectious Diseases Society of America. 2007. Clinical practice guidelines for the management of sporotrichosis: 2007 update by the Infectious Diseases Society of America. Clin Infect Dis 45:1255–1265. doi:10.1086/522765 17968818

[16] Orofino-Costa R, Freitas DFS, Bernardes-Engemann AR, Rodrigues AM, Talhari C, Ferraz CE, Veasey JV, Quintella L, Sousa MSLA de, Vettorato R, Almeida-Paes R de, de Macedo PM. 2022. Human sporotrichosis: recommendations from the Brazilian Society of Dermatology for the clinical, diagnostic and therapeutic management. An Bras Dermatol 97:757–777. doi:10.1016/j.abd.2022.07.001 36155712 PMC9582924

[17] Gremião IDF, Martins da Silva da Rocha E, Montenegro H, Carneiro AJB, Xavier MO, de Farias MR, Monti F, Mansho W, de Macedo Assunção Pereira RH, Pereira SA, Lopes-Bezerra LM. 2021. Guideline for the management of feline sporotrichosis caused by Sporothrix brasiliensis and literature revision. Braz J Microbiol 52:107–124. doi:10.1007/s42770-020-00365-3 32990922 PMC7966609

[18] Viana PG, Gremião IDF, da Silva Antonio IM, Figueiredo ABF, Correa ML, Boechat JS, de Sá Machado AC, de Oliveira RVC, Oliveira MME, Almeida-Paes R, Pereira-Oliveira GR, Pereira SA. 2024. Is terbinafine an effective treatment for feline sporotrichosis? Vet Rec 195:4435. doi:10.1002/vetr.4435 39148234

[19] Thomson J, Trott DJ, Malik R, Galgut B, McAllister MM, Nimmo J, Renton D, Kidd SE. 2019. An atypical cause of sporotrichosis in a cat. Med Mycol Case Rep 23:72–76. doi:10.1016/j.mmcr.2019.01.004 30723664 PMC6350224

[20] Roldán Villalobos W, Monti F, Ferreira T, Sato S, Telles F, Farias M. 2023. Therapeutic efficacy of isavuconazole and potassium iodide in a cat with refractory sporotrichosis. Vet Dermatol 34:624–628. doi:10.1111/vde.13188 37357375

[21] Gremião I.D.F, Miranda LHM, Reis EG, Rodrigues AM, Pereira SA. 2017. Zoonotic epidemic of sporotrichosis: cat to human transmission. PLoS Pathog 13:e1006077. doi:10.1371/journal.ppat.1006077 28103311 PMC5245785

[22] Chaves AR, de Campos MP, Barros MBL, do Carmo CN, Gremião IDF, Pereira SA, Schubach TMP. 2013. Treatment abandonment in feline sporotrichosis - study of 147 cases. Zoonoses Public Health 60:149–153. doi:10.1111/j.1863-2378.2012.01506.x 22898261

[23] Gremião IDF, Miranda L de, Pereira-Oliveira GR, Menezes RC, Machado A de S, Rodrigues AM, Pereira SA. 2022. Advances and challenges in the management of feline sporotrichosis. Rev Iberoam Micol 39:61–67. doi:10.1016/j.riam.2022.05.002 35840526

[24] Veasey JV, Reis APC, Celestrino GA, Silva CE, Santos ES, Mendes DP, Andrade TS, Bonfietti LX, Benard G, Sousa MGT. 2024. Evidence of clinical and laboratory correlation of itraconazole resistance in Sporothrix brasiliensis infection: case report. Microorganisms 12:2132. doi:10.3390/microorganisms12112132 39597522 PMC11596095

[25] Lockhart SR, Ghannoum MA, Alexander BD. 2017. Establishment and use of epidemiological cutoff values for molds and yeasts by use of the clinical and laboratory standards institute M57 standard. J Clin Microbiol 55:1262–1268. doi:10.1128/JCM.02416-16 28202791 PMC5405245

[26] Almeida-Paes R, Oliveira MME, Freitas DFS, Valle ACF do, Gutierrez-Galhardo MC, Zancopé-Oliveira RM. 2017. Refractory sporotrichosis due to Sporothrix brasiliensis in humans appears to be unrelated to in vivo resistance. Med Mycol 55:507–517. doi:10.1093/mmy/myw103 27771622

[27] Espinel-Ingroff A, Abreu DPB, Almeida-Paes R, Brilhante RSN, Chakrabarti A, Chowdhary A, Hagen F, Córdoba S, Gonzalez GM, Govender NP, et al.. 2017. Multicenter, international study of MIC/MEC distributions for definition of epidemiological cutoff values for Sporothrix species identified by molecular methods. Antimicrob Agents Chemother 61:e01057-17. doi:10.1128/AAC.01057-17 28739796 PMC5610517

[28] Nakasu CCT, Waller SB, Ripoll MK, Ferreira MRA, Conceição FR, Gomes ADR, Osório L da G, de Faria RO, Cleff MB. 2021. Feline sporotrichosis: a case series of itraconazole-resistant Sporothrix brasiliensis infection. Braz J Microbiol 52:163–171. doi:10.1007/s42770-020-00290-5 32388779 PMC7966689

[29] Ribeiro Dos Santos A, Gade L, Misas E, Litvintseva AP, Nunnally NS, Parnell LA, Rajeev M, de Souza Carvalho Melhem M, Takahashi JPF, Oliboni GM, Bonfieti LX, Araujo LS, Cappellano P, Venturini J, Lockhart SR, Sexton DJ. 2024. Bimodal distribution of azole susceptibility in Sporothrix brasiliensis isolates in Brazil. Antimicrob Agents Chemother 68:e0162023. doi:10.1128/aac.01620-23 38385701 PMC10989022

[30] Sanchotene KO, Brandolt TM, Klafke GB, Poester VR, Xavier MO. 2017. In vitro susceptibility of Sporothrix brasiliensis: Comparison of yeast and mycelial phases. Med Mycol 55:869–876. doi:10.1093/mmy/myw143 28472490

[31] do Prado CM, Spruijtenburg B, Razzolini E, Chiyo L, Santi C, Martins CA, Santacruz G, Segovia N, Brunelli JP, Cognialli RCR, Meis JF, Vicente VA, de Groot T, Meijer EFJ, Queiroz-Telles F. 2025. Sporothrix brasiliensis treatment failure without initial elevated itraconazole MICs in Felids at Border of Brazil. Emerg Infect Dis 31:1783–1792. doi:10.3201/eid3109.250156 40867021 PMC12407209

[32] Clinical & Laboratory Standards Institution (CLSI). 2017. M38 Reference method for broth dilution antifungal susceptibility testing of filamentous fungi. 3rd edition

[33] Paixão AG, Galhardo MCG, Almeida-Paes R, Nunes EP, Gonçalves MLC, Chequer GL, Lamas C da C. 2015. The difficult management of disseminated Sporothrix brasiliensis in a patient with advanced AIDS. AIDS Res Ther 12:16. doi:10.1186/s12981-015-0051-1 25949269 PMC4422263

[34] Trilles L, Fernández-Torres B, Dos Santos Lazéra M, Wanke B, de Oliveira Schubach A, de Almeida Paes R, Inza I, Guarro J. 2005. In vitro antifungal susceptibilities of Sporothrix schenckii in two growth phases. Antimicrob Agents Chemother 49:3952–3954. doi:10.1128/AAC.49.9.3952-3954.2005 16127080 PMC1195444

[35] Bernardes-Engemann AR, Tomki GF, Rabello VB de S, Almeida-Silva F, Freitas DFS, Gutierrez-Galhardo MC, Almeida-Paes R, Zancopé-Oliveira RM. 2022. Sporotrichosis caused by non-wild type Sporothrix brasiliensis strains. Front Cell Infect Microbiol 12:893501. doi:10.3389/fcimb.2022.893501 35694546 PMC9184675

[36] Rodrigues AM, de Hoog GS, de Cássia Pires D, Brihante RSN, Sidrim JJ da C, Gadelha MF, Colombo AL, de Camargo ZP. 2014. Genetic diversity and antifungal susceptibility profiles in causative agents of sporotrichosis. BMC Infect Dis 14:219. doi:10.1186/1471-2334-14-219 24755107 PMC4021050

[37] Buil JB, Hagen F, Chowdhary A, Verweij PE, Meis JF. 2018. Itraconazole, voriconazole, and posaconazole CLSI MIC distributions for wild-type and azole-resistant Aspergillus fumigatus isolates. J Fungi (Basel) 4:103. doi:10.3390/jof4030103 30158470 PMC6162657

[38] Cañete-Gibas CF, Mele J, Patterson HP, Sanders CJ, Ferrer D, Garcia V, Fan H, David M, Wiederhold NP. 2023. Terbinafine-resistant dermatophytes and the presence of trichophyton indotineae in North America. J Clin Microbiol 61:e0056223. doi:10.1128/jcm.00562-23 37432126 PMC10446870

[39] Teixeira MM, Almeida-Paes R, Bernardes-Engemann AR, Nicola AM, de Macedo PM, Valle ACF, Gutierrez-Galhardo MC, Freitas DFS, Barker BM, Matute DR, Stajich JE, Zancopé-Oliveira RM. 2022. Single nucleotide polymorphisms and chromosomal copy number variation may impact the Sporothrix brasiliensis antifungal susceptibility and sporotrichosis clinical outcomes. Fungal Genet Biol 163:103743. doi:10.1016/j.fgb.2022.103743 36152775

[40] McGinnis MR, Nordoff N, Li RK, Pasarell L, Warnock DW. 2001. Sporothrix schenckii sensitivity to voriconazole, itraconazole and amphotericin B. Med Mycol 39:369–371. doi:10.1080/mmy.39.4.369.371 11556767

[41] Fernández-Silva F, Capilla J, Mayayo E, Guarro J. 2014. Modest efficacy of voriconazole against murine infections by Sporothrix schenckii and lack of efficacy against Sporothrix brasiliensis. Mycoses 57:121–124. doi:10.1111/myc.12112 23879298

[42] CLSI M57. 2024. Principles and Procedures for the Development of Epidemiological Cutoff Values for Antifungal Susceptibility Testing, 1st Edition. Available from: https://clsi.org/standards/products/microbiology/documents/m57. Retrieved 26 Jul 2024.

