# Supplementary material for: Establishment of epidemiological cutoff values for clinically relevant Sporothrix species using CLSI-broth microdilution
Source: Antimicrob Agents Chemother. 2026 Apr 6;70(5):e01907-25. doi: 10.1128/aac.01907-25 (PMC13148023; doi:10.1128/aac.01907-25)
Supplement: Table S1 — List of the 19 laboratories sending MIC data in the five continents. [file aac.01907-25-s0001.docx]

**Supplementary Table. List of the 19 laboratories sending MIC data in the five continents**

| Continent | Country | Labs | Laboratory Name |
| --- | --- | --- | --- |
| Asia | India | 1 | Mycology Division, Department of Medical Microbiology, Post Graduate Institute of Medical Education & Research |
|  | India | 2 | National Reference Laboratory for Antimicrobial Resistance in Fungal Pathogens, Vallabhbhai Patel Chest Institute, University of Delhi |
|  | China | 3 | Peking University First Hospital |
|  |  |  |  |
| Europe |  | 4 | Radboudumc-CWZ Center of Expertise for Mycology, Canisius-Wilhelmina Hospital (CWZ) |
|  | United Kingdom | 5 | National Mycology Reference Laboratory |
|  |  |  |  |
| North America | Canada | 6 | Laboratoire de santé publique du Québec, Institut national de santé publique du Québec (INSPQ) |
|  | United States | 7 | Johns Hopkins University |
|  | United States | 8 | Mycotic Diseases Branch, Centers for Disease Control and Prevention |
|  | Mexico | 9 | Universidad Autónoma de Nuevo León |
|  |  |  |  |
| South America | Argentina | 10 | Mycology Department of INEIA ANLIS “Dr. C. G. Malbran” |
|  | Argentina | 11 | Universidad Nacional del Litoral |
|  | Brazil | 12 | Universidade Federal do Paraná (UFPR) |
|  | Brazil | 13 | Laboratório de Pesquisa Clínica em Dermatozoonoses em Animais Domésticos (Lapclin-Dermzoo), Instituto Nacional de Infectologia Evandro Chagas (INI), Fiocruz |
|  |  | 14 | Universidade Federal de São Paulo (UNIFESP) |
|  | Brazil | 15 | Centro de Investigações em Micologia Médica (CIMM), Universidade Federal do Espírito Santo (UFES) |
|  | Brazil | 16 | Parasitology and Mycology Center, Instituto Adolfo Lutz |
|  | Brazil | 17 | Laboratório de Biologia Celular de Fungos (LBCF) at Universidade Federal do Rio de Janeiro (UFRJ) |
|  | Brazil | 18 | One Health Microbiology Laboratory (OneHMic) at Universidade Federal do Ceará (UFC) |
|  |  |  |  |
| Oceania | Australia | 19 | National Mycology Reference Centre, SA Pathology, Adelaide, Australia |
